# Genome-based species-specific primers for rapid identification of six species of *Lactobacillus acidophilus* group using multiplex PCR

**DOI:** 10.1371/journal.pone.0230550

**Published:** 2020-03-20

**Authors:** Inhwan You, Eun Bae Kim

**Affiliations:** 1 Department of Animal Life Science, College of Animal Life Sciences, Kangwon National University, Chuncheon, Republic of Korea; 2 Institute of Animal Resources, Kangwon National University, Chuncheon, Republic of Korea; National Centre For Cell Science, INDIA

## Abstract

Many *Lactobacillus* species are frequently isolated from dairy products, animal guts, and the vaginas of healthy women. However, sequencing-based identification of isolated *Lactobacillus* strain is time/cost-consuming and lobor-intensive. In this study, we developed a multiplex PCR method to distinguish six closely related species in the *Lactobacillus acidophilus* group (*L*. *gasseri*, *L*. *acidophilus*, *L*. *helveticus*, *L*. *jensenii*, *L*. *crispatus*, and *L*. *gallinarum*), which is based on species-specific primer sets. Altogether, 86 genomes of 9 *Lactobacillus* species from the National Center of Biotechnology Information (NCBI) database were compared to detect species-specific genes and design six species-specific primer sets. The PCR conditions of the individual primer sets were optimized via gradient PCR methods. A final multiplex PCR condition was also optimized for a mixture of all six primer sets mixed. When identifying a single strain, the optimized multiplex PCR method can specifically detect one of the six species, but no band was amplified at least from the other *Lactobacillus* and *Enterococcus* species. These results indicated that species-specific primer sets designed from the genome comparison could identify one strain within the six *Lactobacillus* species by a single PCR reaction. Using the method described here, we will be able to save time, cost, and labor during species identification and screening of commercially important probiotic lactobacilli.

## Introduction

Lactic acid bacteria (LAB) are of great economic importance to the food biotechnology sector as well as in the production of other health supplements [1–5]. Lactobacilli are one of the frequently used commercial probiotic groups within LAB and are considered to play a beneficial role in the human and animal intestinal tract [6–9].

The *Lactobacillus acidophilus* homology group is a clade of homologous *Lactobacillus* species categorized using molecular-based phenotypic and genotypic techniques [10,11]. Species within the same homology group share similar genomic patterns; however, phenotypes and functions are species dependent [12,13]. Hence, there is a growing interest towards bacterial characteristic identification at the species and strain level. Although there are several methods to identify bacteria via phenotypes and biochemical characteristics, they are often ambiguous and steeped in uncertainty.

Molecular methods based on 16S ribosomal DNA gene sequencing are widely used to identify bacteria at the species level [14–17]. However, the *L*. *acidophilus* group is genotypically closely related and these sequences are highly similar among species. Therefore, a reliable and rapid method is needed to classify the *L*. *acidophilus* group. In the present study, we investigated species-specific primer sets that were used to identify 6 species of *L*. *acidophilus* group (*L*. *gasseri*, *L*. *acidophilus*, *L*. *helveticus*, *L*. *jensenii*, *L*. *crispatus*, *L*. *gallinarum*) via comparative genomics.

## Materials and methods

### Phylogenetic analysis of 16S rRNA sequences

The 16S rRNA gene sequence similarity of 11 *Lactobacillus* species including *L*. *acidophilus* group was verified using *in silico* analysis. The sequences were downloaded from NCBI and phylogenetic analysis with the neighbor joining method via MEGA7 software [18] with a 1000 bootstrap analysis was performed. The percentage of sequence identity was created by Clustal Omega after aligned using the MEGA7.

### Genome collection and ortholog detection

Altogether, 86 genomes of 9 different *Lactobacillus* species (*L*. *acidophilus*, *L*. *amylovorus*, *L*. *crispatus*, *L*. *gasseri*, *L*. *gallinarum*, *L*. *helveticus*, *L*. *jensenii*, *L*. *johnsonii*, and *L*. *delbrueckii*) were obtained from the NCBI database (S1 Table). Genome sequences were annotated using the RAST Server [19] with default parameters for bacteria. The protein-coding sequences (CDS) from the annotation were mutually aligned with similar CDS identity (parameters of ≥ 65%) using Global Alignment Short Sequence Search Tool (GASSST) [20]. Overall, 69,318 ortholog groups were detected from nine *Lactobacillus* species.

### Species-specific gene selection and primer pair design

For the determination of species-specific gene, all 86 genomes were screened for the presence of each representative ortholog. Contig sequences were fragmented into 50 bp reads at intervals of 7 bp and aligned with the representative orthologues using the GASSST software (sequence similarity of ≥ 65%). The fragmented reads that demonstrated low coverage (≤ 30%) of the full length of each ortholog were withdrawn. The species-specific gene was determined based on the coverage rate (%) and later used to design the primer sets via the Primer 3 Plus software [21]. All primer sets were synthesized from Bioneer (Daejeon, South Korea).

### Bacterial strains

To ensure that the individual primer sets were adequate for target species detection, 11 bacterial strains were used. Six bacterial strains were obtained from the Korean Agricultural Culture Collection (KACC), one from the Korean Collection for Type Cultures (KCTC). *Lactobacillus reuteri* KLR3004 (GenBank accession NO. MIMT00000000), *L*. *salivarius* 144, *L*. *plantarum* CJLP133 (GenBank accession NO. GQ336971) and *Enterococcus faecalis* JB00072 were isolated locally and identified using 16S rRNA sequencing (Table 1.)

**Table 1:** Bacterial strains used for the multiplex PCR optimization.

Species	Strains	Origins
*Lactobacillus gasseri*	KACC 12424	Unknown
*Lactobacillus acidophilus*	KACC 12419	Human
*Lactobacillus helveticus*	KACC 12418	Undefined natural whey starter
*Lactobacillus jensenii*	KACC 12437	Human vaginal discharge
*Lactobacillus crispatus*	KACC 12439	Human vaginal
*Lactobacillus gallinarum*	KACC 12370	Chicken, crop
*Lactobacillus reuteri*	KLR3004	Pig feces
*Lactobacillus salivarius*	144	Local isolated from piglet
*Lactobacillus plantarum*	CJLP133	Korea kimchi
*Enterococcus faecalis*	JB00072	Cheese
*Enteroccous faecium*	KCTC13225	Unknown

### Genomic DNA extraction

The bacterial strains were incubated in De Man, Rogosa and Sharpe (MRS) broth (MB Cell, South Korea) at 37°C for 24 h. The cells, obtained after centrifugation at 13,000 rpm for 1.5 min, were washed twice with 0.85% NaCl. Genomic DNA was then extracted from the washed cells using G-spin Genomic DNA Extraction Kit for bacteria (iNtRON Biotechnology, South Korea) following the manufacturer’s instructions. DNA concentration and purity were determined at an absorbance ratio of 260/280 nm using a Nanodrop (Titertek-Berthold, Germany).

### Development of multiplex PCR conditions

The PCR reaction mixture, containing 0.8 mM dNTPs, 1.25 mM MgCl_2_, 1.5 U of i-*Taq* DNA polymerase (iNtRON Biotechnology, South Korea), 10× PCR buffer (2 μL, 20 mM MgCl_2_), and about 5 ng/μL of template DNA, was adjusted to 20 μL with sterile water. The primer sets were added 2.5 pmol each for *L*. *gasseri*, *L*. *helveticus*, and *L*. *jensenii*; 5 pmol for *L*. *crispatus*; and 10 pmol for *L*. *acidophilus* and *L*. *gallinarum* (forward and reverse, each). To optimize the multiplex PCR conditions, gradient PCR method with various annealing temperatures was tested twice to obtain precise conditions (from 54°C to 64°C and from 58°C to 63°C). The PCR reaction mixture conditions were as follows: initial denaturation at 94°C for 5 min followed by 40 cycles of amplification (denaturation at 94°C for 20 s, annealing at 63°C for 30 s, and extension at 72°C for 1.5 min) and a final extension step at 72°C for 7 min. The amplified products were then run on a 1.5% agarose gel with TAE buffer containing ethidium bromide and visualized using a Gel Documentation System (Bio-Rad, USA).

## Results

### *Lactobacillus* 16S rRNA sequence comparison

Members of the *Lactobacillus acidophilus* group demonstrated that their sequences were similar and formed one branch (S1 Fig). The sequence similarity of the six target species varied between 92.12–99.17%. The most closely related groups were *L*. *gallinarum* and *L*. *helveticus* with 99.17% similarity (Table 2). These results indicated that the members of the *Lactobacillus acidophilus* group were difficult to identify using only 16S rRNA sequences, thus requiring more sensitive detection methods.

**Table 2:** 16S rRNA sequence identity percentage of six species of the *Lactobacillus acidophilus* group.

Strains	*L*. *helveticus*	*L*. *gallinarum*	*L*. *acidophilus*	*L*. *crispatus*	*L*. *jensenii*	*L*. *gasseri*
***L*. *helveticus***	100.00	99.17	98.35	98.41	92.28	92.69
***L*. *gallinarum***	99.17	100.00	98.28	98.22	93.05	92.88
***L*. *acidophilus***	98.35	98.28	100.00	98.41	92.67	92.31
***L*. *crispatus***	98.41	98.22	98.28	100.00	93.05	92.12
***L*. *jensenii***	92.28	93.05	92.67	92.69	100.00	94.01
***L*. *gasseri***	92.69	92.88	92.69	92.69	92.69	100.00

All strains are type strains; *L*. *helveticus* ATCC 15009, *L*. *gallinarum* ATCC 33199, *L*. *acidophilus* ATCC 4356, *L*. *crispatus* ATCC 33820, *L*. *jensenii* ATCC 25258, L. *gasseri* ATCC 33323. Each numbers indicated sequence identity percentage.

### Selection of target genes

Among 69,318 orthologues from the 86 genomes, several unique genes were found to exist in *L*. *gasseri*, *L*. *acidophilus*, *L*. *helveticus*, *L*. *jensenii*, *L*. *crispatus*, and *L*. *gallinarum* in the genome database. While hypothetical proteins were deduced from *L*. *gasseri*, *L*. *crispatus*, and *L*. *gallinarum*, the major facilitator superfamily (MFS)-transporter from *L*. *acidophilus*, acyl carrier protein (ACP) S-malonyltransferase from *L*. *helveticus*, and acetoacetate decarboxylase from *L*. *jensenii* were used for further analysis. Each gene contained suitable sequences for the design of specific primer pairs for the six species (Table 3).

**Table 3:** Target genes and primers used in this study.

		Primers			
Gene	Species	Pair	Sequence (5'-3')	Product size (bp)	Tm
Hypothetical protein	*L*. *gasseri*	F	AATACTCCCGAAGCACGTCA	1241	58.4
		R	TCATTGTGTTTGGCAATCGT		54.3
MFS-transporter	*L*. *acidophilus*	F	TCATGTTGGGATGCAATGAG	828	56.4
		R	TTTCAAAACTTGTCCTGCTG		54.3
ACPS-malonyltransferase	*L*. *helveticus*	F	GTATGATCGTTCGCCACCAC	680	60.5
		R	ATTGTCGCCATGAGTACAGG		58.4
Acetoacetate decarboxylase	*L*. *jensenii*	F	ATGCTTGGCGCTTATCCTT	540	55.2
		R	ATATGGTGCGATTTCATCTGG		57.4
Hypothetical protein	*L*. *crispatus*	F	TGGCGAAGAGACACCAATATC	376	59.4
		R	TGACGTAACGCATGATGAAT		54.3
Hypothetical protein	*L*. *gallinarum*	F	AGTCTTGAGCCCGTAAAAGC	224	58.4
		R	TTGCCAAACGGTTCTTCTTT		54.3

### PCR conditions optimization of individual species-specific primer pairs

**Fig 1:**
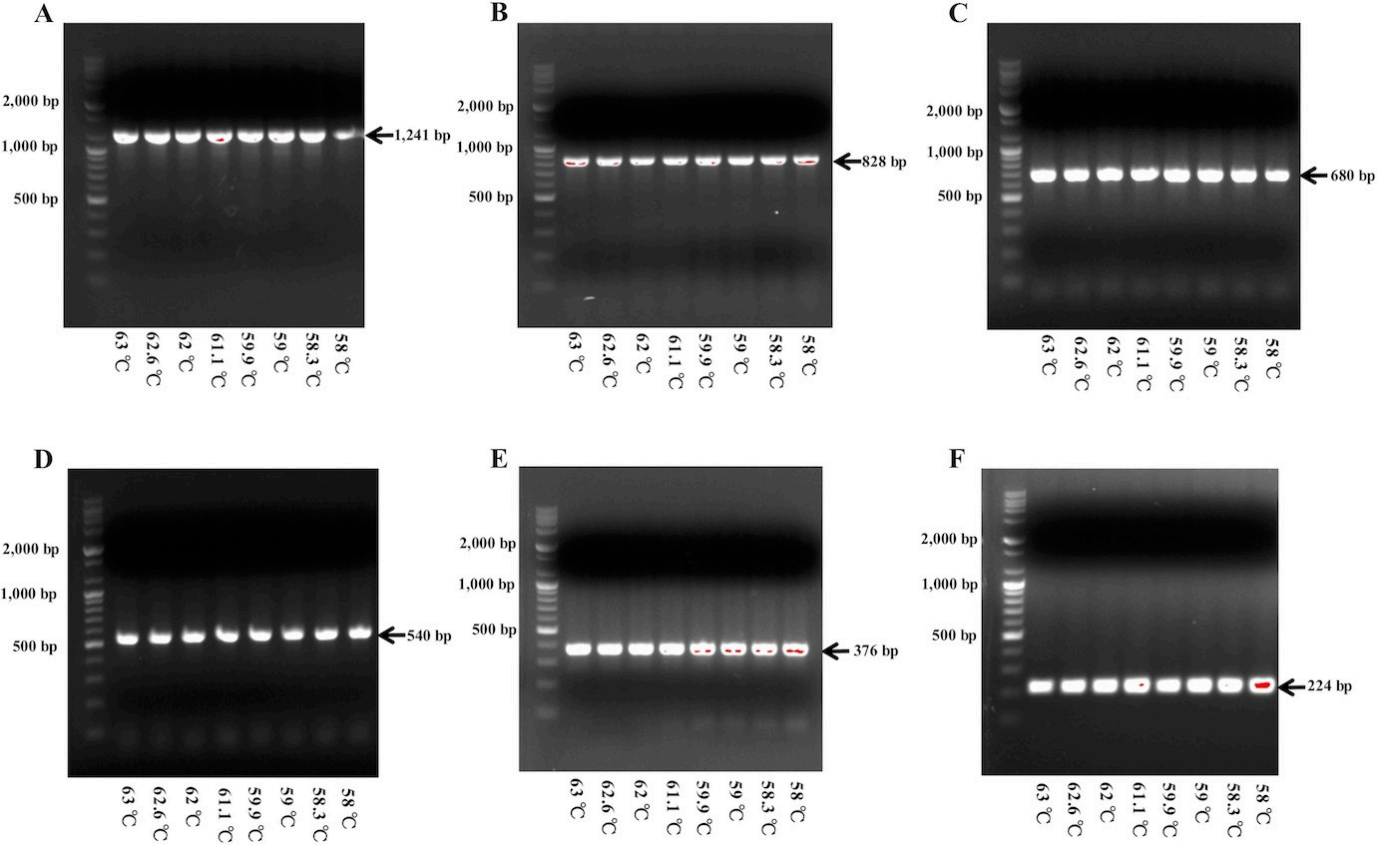
Gel electrophoresis results of gradient PCR products amplified with individual primer sets. A, *Lactobacillus gasseri*; B, *Lactobacillus acidophilus*; C, *Lactobacillus helveticus*; D, *Lactobacillus jensenii*; E, *Lactobacillus crispatus*; F, *Lactobacillus gallinarum*; M, size marker(bp); 1, 63°C; 2, 62.6°C; 3, 62°C; 4, 61.1°C; 5, 59.9°C; 6, 56°C; 7, 58.3°C; 8, 58°C.

The quantities of all the primer sets were adjusted to output a similar intensity when viewed after gel electrophoresis. The annealing temperature of individual primer sets ranged from 58°C to 63°C using gradient PCR method. The results were visualized after gel electrophoresis and the annealing temperature range demonstrated clear bands (Fig 1). The validated annealing temperature was used for further multiplex PCR condition optimization.

### Re-optimization of multiplex PCR conditions with six mixed primer sets

**Fig 2:**
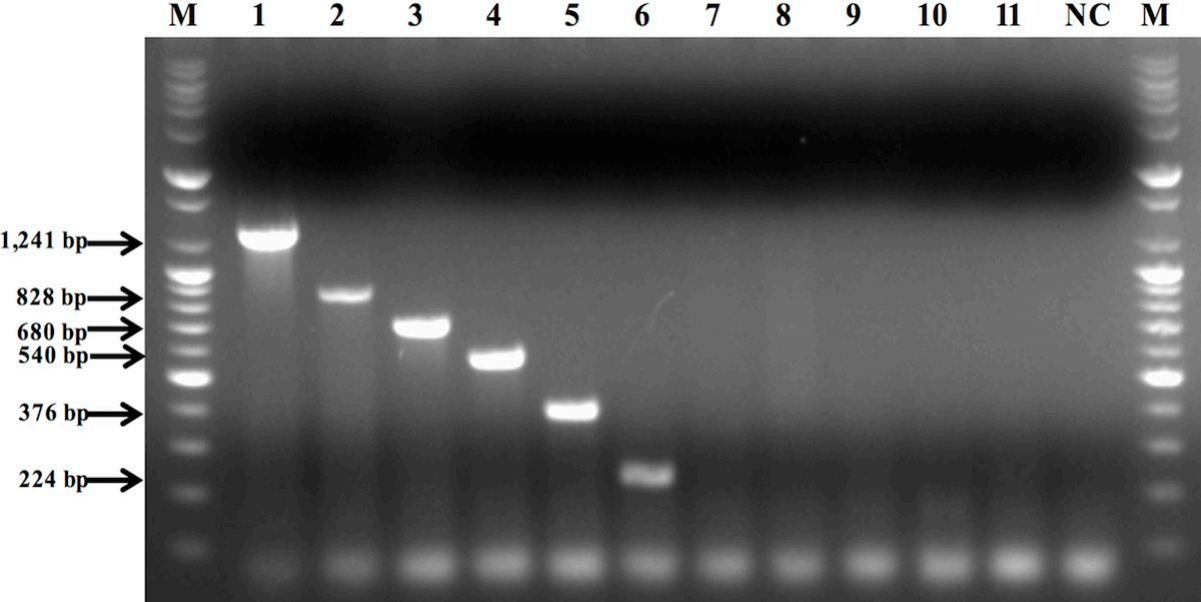
Comparison of six species-specific primer sets with other bacterial genus and species. All primer sets were pooled for the multiplex PCR reaction. M, size marker(bp); 1, *Lactobacillus gasseri*; 2, *Lactobacillus acidophilus*; 3, *Lactobacillus helveticus*; 4, *Lactobacillus jensenii*; 5, *Lactobacillus crispatus*; 6, *Lactobacillus gallinarum*; 7, *Lactobacillus plantarum*; 8, *Lactobacillus salivarius*; 9, *Lactobacillus reuteri*; 10, *Enterococcus faecalis*; 11, *Enterococcus faecium*; NC, negative control.

Since low annealing temperatures can form multiple bands, the highest temperature (63°C) from the gradient PCR was selected. Next, the six primer sets were mixed for multiplex PCR at the annealing temperature of 63°C. To verify the species-specific detection ability of the primer sets, other species (*L*. *plantarum*, *L*. *salivarius*, and *L*. *reuteri*) and genera (*E*. *faecalis* and *E*. *faecium)* were used as negative controls. The amplicon products were examined using a 1.5% agarose gel. Only six species were detected on the gel electrophoresis (Fig 2). Each target species was distinguishable by band size on the gel because the molecular weights of the PCR products were identical to the theoretical values (1,241 bp for *L*. *gasseri*, 828 bp for *L*. *acidophilus*, 680 bp for *L*. *helveticus*, 540 bp for *L*. *jensenii*, 376 bp for *L*. *crispatus*, and 224 bp for *L*. *gallinarum*).

## Discussion

PCR-based species identification is one of the most important, time-efficient, and reliable tools available for detecting bacteria. Hence, species identification is key in developing and selecting bacterial species for specific industrial applications. However, the close genetic relationship among the species makes it difficult to accurately identify bacterial strains. Selecting a unique gene provides clues that physiological differences between particular microorganism and others. These clues allow to understand the species-specific characteristics, for example, habitat, essential nutrients and environmental applicability [22–25]. Unique gene can be used to identify specific microorganisms or whether a particular microorganism exists in a complex sample [25–28]. Therefore, selecting species/strain-specific gene can be used to bacterial identification by understanding unique characteristic of the species/strain. In addition, a selected gene can be applied in the quantitative analysis of microorganisms via qPCR in microorganism with species specificity [29, 30] and it will be a useful tool in gut-microorganisms/ fermented food study.

Previously, PCR-based reports with regards to identifying Lactobacilli occurred mostly at the genus level, and only limited genes such as 16S rRNA were used at the species level [31–34]. In the present study, we designed species-specific primer sets based on genome comparison to detect six species within the *L*. *acidophilus* group and successfully developed a multiplex PCR method for a mixture of all the six primer sets to do so. For the genome comparison, whole genomes were used not only 16S rRNA gene sequences because of highly similar among *L*. *acidophilus* group as we represented in Table 2. We obtained species-specific genes for each species by comparing whole genomes, these genes could complement and reinforce other detection methods that are difficult to classify using only 16S rRNA gene.

To investigate detection ability on a complex bacterial sample, DNA of all strains used in this study was mixed (6 target species and 5 non-target species). Although single targets were successfully detected, mixed DNA was not effective for our present method. When DNA were mixed, only five species were identified, but only one species (*L*. *acidophilus*) was not (S2 Fig). As we could not fully understand why it was not successful, further investigation is needed. Furthermore, to develop a multiplex PCR method for complex bacterial samples such as feces and fermented foods requires a much more complex bioinformatic analysis because we have to consider much more species.

As mentioned previously, unique genes existed in each of the six species i.e., the three different hypothetical proteins in *L*. *gasseri*, *L*. *crispatus*, and *L*. *gallinarum*; MFS-transporter in *L*. *acidophilus*; ACP S-malonyltransferase in *L*. *helveticus*; and acetoacetate decarboxylase in *L*. *jensenii*. MFS is the largest group of solute transporters that transports different molecules such as sugars, amino acids, and vitamins. Although the MFS-transporter families are quite different from one another, their sequence similarity within families is highly significant [35]. ACP S-malonyltransferase is an essential enzyme that initiates fatty-acid biosynthesis in bacteria [36–38]. Acetoacetate decarboxylase is essential for solvent production and catalyzing the decarboxylation of acetoacetate to acetone [39,40]. However, these genes also exist in some other strains of the *L*. *acidophilus* group (MFS-transporter exists in *L*. *ultunensis* and *L*. *kefiranofaciens*; ACP S-malonyltransferase exists in *L*. *gallinarum* and *L*. *crispatus*; and acetoacetate decarboxylase exists in *L*. *psittaci* and *L*. *salivarius*). *In silico* analysis showed that these genes that are present in different species differed in sequence even though they had similar functions. *In vitro* experiments are required to confirm whether the primer pairs designed in this study are still species-specific even when other species that are not covered in this study and have the same gene are included. The six species-specific genes found in this study may have unique or alternative functions in the respective species. Further research is needed to define the role of specific genes in each species.

The successful and specific identification of a majorly recognized probiotic group in this study demonstrates the capability of multiplex PCR using species-specific primer pairs in single bacterial identification without the need for sequencing processes with obvious applications in industry and research. Since our method was developed using currently known genomes, it may not be valid if novel strains are found. Therefore, further studies need to be carried out on the genomes of bacterial strains.

## Supporting information

S1 Table *Lactobacillus* species isolates and genomes used in this study. (PDF)

S2 Table *Lactobacillus* species gene identity with selected orthologs. (PDF)

S3 Table Quantities of PCR components used in this study. (PDF)

S1 Fig Phylogenetic tree of the *Lactobacillus* 16S rRNA gene sequences. (PDF)

S2 Fig Gel electrophoresis results of mixed genomic DNA containing target and untargeted species. (PDF)

S1 Data Species-specific gene sequences for each species. (PDF)

S2 Data Sequences of PCR products of each species. (PDF)
