# Supplementary material for: Genome-based species-specific primers for rapid identification of six species of Lactobacillus acidophilus group using multiplex PCR
Source: PLoS One. 2020 Mar 20;15(3):e0230550. doi: 10.1371/journal.pone.0230550 (PMC7083307; doi:10.1371/journal.pone.0230550)
Supplement: S1 Table — (PDF) [file pone.0230550.s001.pdf]

S1 Table. *Lactobacillus* species isolates and genomes used in this study.

Species	Strain No.	GenBank accession No.	Genome size (Mb)	Isolation origin(s)
Lactobacillus acidophilus	ATCC 4356	JRUT01000000	1.95	Homo sapiens
	ATCC 4796	ACHN00000000	2.02	Unknown
	CFH-AYUA01	AYUA01000000	1.25	Yogurt starter culture
	CFH-AYUB01	AYUB00000000	1.97	Yogurt starter culture
	CIP76.13	CBLQ000000000	2.02	Human
	CIRM-BIA 442	CBLP000000000	1.98	Dairy products
	CIRM-BIA 445	CBLR000000000	2	Dairy products
	DSM 20242	CBLT000000000	2.04	Unknown
	DSM 9126	CBLS000000000	1.99	Unknown
	FSI4	CP010432	1.99	Yogurt
	JCM 1132	BALR00000000	1.95	Unknown
	La-14	CP005926	1.99	Commercial dietary supplement
	NCFM	CP000033	1.99	Human
Lactobacillus amylovorus	30SC	CP002559	2.09	Swine intestine
	GRL 1112	CP002338	2.12	Porcine feces
	GRL 1118	CP002609	1.97	Pig Ileum
Lactobacillus crispatus	125-2-CHN	ACPV00000000	2.3	Vaginal isolate from a healthy Chinese woman
	2029	AVFH00000000	2.19	Genital tract of a healthy woman
	214-1	ADGR00000000	2.06	Human vaginal
	CTV-05	ADML00000000	2.36	Human vaginal
	EM-LC1	AXLM00000000	1.82	Homo sapiens facal
	FB049-03	AGZF00000000	2.45	Human vaginal
	FB077-07	AGZG00000000	2.69	Human vaginal

	JV-V01	ACKR00000000	2.22	Human vaginal
	MV-1A-US	ACOG00000000	2.25	Vaginal isolate from a healthy US woman
	MV-3A-US	ACQC00000000	2.43	Vaginal isolate from a healthy US woman
	SJ-3C-US	ADDT00000000	2.16	Vaginal isolate from a healthy US woman
	ST1	FN692037	2.04	Chicken isolate
Lactobacillus delbrueckii subsp. bulgaricus	2038	CP002341	1.87	Naturally fermented yak milk in Qinghai
	ATCC BAA-365	CP000412	1.85	Derived from existing strain
	ATCC 11842	CR954253	1.86	Bulgarian yogurt
	CCET01	CCET00000000	1.85	Unknown
	CCEU01	CCEU00000000	1.81	Unknown
	CNCM I-1519	AGHW00000000	1.79	Human fecal
	CNCM I-1632	AGFO00000000	1.76	Human fecal
	JXRV01	JXRV00000000	2.06	Yogurt
	ND02	CP002341	2.13	Naturally fermented yak milk in Qinghai
	PB2003/044-T3-4	AEAT00000000	1.97	Human
Lactobacillus delbrueckii subsp. Delbrueckii	DSM 20074	AZCR00000000	1.95	Sour grain mash
Lactobacillus delbrueckii subsp. Jakobsenii	DSM 26046	ALPY00000000	1.73	Malted sorghum wort
Lactobacillus delbrueckii subsp. lactis	CCDT01	CCDT00000000	1.91	Unknown
	CCDS01	CCDS00000000	2.05	Unknown
	CCDU01	CCDU00000000	2.08	Unknown
	CCDV01	CCDV00000000	2.1	Unknown
	CRL581	ATBQ00000000	2.13	Argentinian hard cheese

	DSM 20072	AZDE00000000	2.16	Emmental cheese
Lactobacillus gallinarum	DSM 10532	AZEL00000000	1.93	Chicken crop
Lactobacillus gasseri	130918	NZ_CP006809	1.93	Infant feces
	2016	AUUE00000000	1.87	Vaginal tract of a healthy female
	202-4	ACOZ00000000	1.82	Unknown
	224-1	ADFT00000000	2	Unknown
	ATCC 33323	BALQ00000000	1.89	Human
	CECT 5714	AKFQ00000000	1.9	Human milk
	JV-V03	NZ_ACGO00000000	2.01	Unknown
	MV.22	ABWH00000000	1.93	Human vaginal tract
	SJ.9E.US	ADDU00000000	1.78	Unknown
	SV.16A.US	ADDY00000000	2	Unknown
Lactobacillus helveticus	ATCC 10386	JRQG00000000	1.88	Dairy product
	CIRM-BIA 101	CBUN000000000	2.07	Milk product
	CIRM-BIA 103	CBUM000000000	2	Milk product
	CIRM-BIA 104	CBUL000000000	2.06	Milk product
	CIRM-BIA 951	CBUK000000000	1.9	Milk product
	CIRM-BIA 953	CBUH000000000	2.38	Milk product
	CNRZ32	CP002081	2.22	Commercial cheese starter
	DPC 4571	CP000517	2.08	Swiss cheese
	DSM 20075	ACLM00000000	2.02	Unknown
	H10	CP002429	2.17	Traditional fermented milk in Tibet
	H9	CP002427.1	1.87	Kurutin Tibet
	KLDS1.8701	CP009907	2.1	Traditional cheese in Sinkiang
	M3	JRTS00000000	1.85	Bacteriophage Insensitive Mutant of strain ATCC 10386
	MB2-1	AEYL01000001.1	2.08	Traditional Sayram ropy fermented milk

	MTCC 5463	AEYL00000000	2.04	Healthy human vaginal cavity
	R0052	CP003799	2.12	Sweet acidophilus milk
Lactobacillus jensenii	115-3-CHN	ACQN00000000	1.64	Unknown
	1153	ABWG00000000	1.74	Unknown
	269-3	ACOY00000000	1.68	Unknown
	27-2-CHN	ACOF00000000	1.63	Unknown
	JV-V16	CM000953	1.6	Unknown
	MD IIE-70(2)	AVCU00000000.	1.75	Genital tract of a healthy woman
	SJ-7A-US	ACQD00000000	1.72	Unknown
Lactobacillus johnsonii	ATCC 33200	ACGR00000000	1.78	Human blood
	DPC 6026	CP002464	1.96	Porcine small intestine
	FI9785	FN298497	1.78	Poultry
	N6-2	CP006811	1.88	Type 1 diabetes-resistant rat model
	NCC 533	AE017198	1.99	Human
	pf01	AFQJ00000000	1.97	Piglet feces
