# Supplementary material for: Genome-based species-specific primers for rapid identification of six species of Lactobacillus acidophilus group using multiplex PCR
Source: PLoS One. 2020 Mar 20;15(3):e0230550. doi: 10.1371/journal.pone.0230550 (PMC7083307; doi:10.1371/journal.pone.0230550)
Supplement: S2 Table — (PDF) [file pone.0230550.s002.pdf]

S3 Table. Alignment coverage for presence of selected orthologs.

		Ortholog ID (Sequence coverage percentage)							
**Species**	**Strains**	**50484(%)**	**20071(%)**	**19694(%)**	**50754(%)**	**8590(%)**	**34479(%)**	**3614(%)**	**47234(%)**
Lactobacillus acidophilus	ATCC 4356	100	0	0	18.8	6.9	0	0	5.5
	ATCC 4796	100	0	0	20.8	15.3	0	0	10.2
	CFH-AYUA01	100	0	0	0	12.9	0	0	5.8
	CFH-AYUB01	100	0	0	18.4	7.8	0	0	5.4
	CIP76.13	100	0	0	18.8	6.9	0	0	5.5
	CIRM-BIA 442	100	0	0	19.2	7.8	0	9.3	10.2
	CIRM-BIA 445	100	0	0	18.4	0	0	0	5.9
	DSM 20242	100	0	0	18.8	7.8	0	8.9	10.2
	DSM 9126	100	0	0	18.4	6.9	0	0	5.5
	FSI4	100	0	0	18.4	0	0	0	5.9
	JCM 1132	100	0	0	18.8	7.8	0	8.9	9.6
	La-14	100	0	0	20.8	11	0	0	10.2
	NCFM	100	0	0	18.6	6.9	0	8.9	5.5
Lactobacillus amylovorus	30SC	0	100	0	0	0	0	0	0
	GRL 1112	0	100	0	0	0	0	12	0
	GRL 1118	0	100	0	0	0	0	11	4.6
Lactobacillus crispatus	125-2-CHN	0	0	100	0	14.8	0	0	12.9
	2029	0	5.9	100	0	7.7	0	0	0
	214-1	0	5.9	100	20.4	9.2	0	0	13.3
	CTV-05	0	5.9	100	0	14.1	0	0	9.8
	EM-LC1	0	5.9	100	0	0	0	0	4.9

	FB049-03	0	11.9	100	0	9.9	0	0	4.6
	FB077-07	0	6	100	20.4	7.7	9.7	0	12.8
	JV-V01	0	5.9	100	27.1	0	0	9	4.4
	MV-1A-US	0	0	100	16.8	0	0	0	14.8
	MV-3A-US	0	6.7	100	0	7.9	0	9	4.6
	SJ-3C-US	0	0	100	0	7.8	0	0	19.7
	ST1	0	0	100	13.7	7.7	0	0	4.7
Lactobacillus delbrueckii subsp. bulgaricus	2038	9.3	5.6	0	0	0	0	0	0
	ATCC BAA-365	9.8	0	0	0	0	0	0	0
	ATCC 11842	9.9	5.5	0	0	0	0	0	0
	CCET01	10.4	5.6	0	0	0	0	0	0
	CCEU01	8.6	0	0	0	0	0	0	0
	CNCM I-1519	10.6	5.7	0	0	5.4	0	0	0
	CNCM I-1632	10	0	0	0	0	0	0	0
	JXRV01	9.4	0	0	0	0	0	0	0
	ND02	8.6	0	0	0	0	8.1	0	0
	PB2003/044-T3-4	9.8	0	0	0	0	0	0	0
Lactobacillus delbrueckii subsp. Delbrueckii	DSM 20074	9.1	0	0	0	0	0	0	0
Lactobacillus delbrueckii subsp. Jakobsenii	DSM 26046	10	5.6	0	0	0	0	0	0
Lactobacillus delbrueckii subsp. lactis	CCDT01	9.8	5.5	0	0	0	0	0	0
	CCDS01	10.7	0	0	0	0	10.3	0	0
	CCDU01	9.8	0	0	0	0	10.8	9.2	0
	CCDV01	9.8	0	0	0	0	8.2	0	0
	CRL581	10.7	0	11.9	0	0	10.4	0	0

	DSM 20072	0	0	0	0	0	9	0	0
Lactobacillus gallinarum	DSM 10532	0	0	0	100	7.7	0	0	6.2
Lactobacillus gasseri	130918	0	5.6	0	13	99.9	0	0	87.4
	2016	8.2	5.6	0	0	100	0	0	8.1
	202-4	0	5.7	0	13.9	100	0	0	8.1
	224-1	8.2	5.7	0	27.8	100	0	0	5.7
	ATCC 33323	8.9	0	0	13.9	100	0	9.2	8.1
	CECT 5714	8.2	5.7	0	11	100	0	0	10.4
	JV-V03	0	5.6	0	13	100	0	0	90.8
	MV.22	8.9	0	0	14.3	100	0	9.2	5.2
	SJ.9E.US	8.2	5.6	0	14.3	99.9	0	9.2	5.3
	SV.16A.US	8.2	0	0	27.3	100	8.8	0	8
Lactobacillus helveticus	ATCC 10386	0	6.2	0	26	22.8	100	0	0
	CIRM-BIA 101	0	11.6	0	0	16.9	100	0	0
	CIRM-BIA 103	0	5.7	0	0	22.7	100	0	4.4
	CIRM-BIA 104	0	6.2	0	0	7.6	100	0	11.3
	CIRM-BIA 951	0	6.5	0	8.5	9.6	100	0	0
	CIRM-BIA 953	0	6.1	26.4	15.4	15.7	100	0	0
	CNRZ32	0	0	0	0	7.6	100	0	9.4
	DPC 4571	0	6.5	24.2	0	11	100	0	5.4
	DSM 20075	0	5.6	0	0	8.4	100	0	4.3
	H10	0	5.8	28.5	29.3	9.6	100	0	5.4
	H9	0	0	0	13.9	7.6	100	0	9
	KLDS1.8701	0	5.3	0	0	7.6	100	0	18.9
	M3	0	6.3	0	0	8.3	100	0	4.6

	MB2-1	0	5.7	0	15.2	14.9	100	0	4.5
	MTCC 5463	0	0	0	11.8	14.9	100	0	0
	R0052	0	6.2	29	0	16.8	100	0	11.7
Lactobacillus jensenii	115-3-CHN	0	0	0	0	0	0	100	0
	1153	0	5.5	0	14.1	0	0	100	0
	269-3	0	0	0	12.8	7.3	0	100	0
	27-2-CHN	0	5.9	0	14.1	22.9	0	100	4.6
	JV-V16	0	0	0	13.9	8	0	100	4.7
	MD IIE-70(2)	0	0	0	13	17.1	0	100	0
	SJ-7A-US	0	0	0	0	7.3	0	100	0
Lactobacillus johnsonii	ATCC 33200	0	0	0	20.3	20.4	0	0	100
	DPC 6026	0	0	0	19.2	9.6	0	0	100
	FI9785	8.8	6	0	0	7.9	0	0	100
	N6-2	0	0	0	15.9	19.2	0	0	100
	NCC 533	0	0	0	17.4	25.5	0	0	100
	pf01	9.6	0	0	20.3	9.6	0	0	100
