# Supplementary material for: Genome-based species-specific primers for rapid identification of six species of Lactobacillus acidophilus group using multiplex PCR
Source: PLoS One. 2020 Mar 20;15(3):e0230550. doi: 10.1371/journal.pone.0230550 (PMC7083307; doi:10.1371/journal.pone.0230550)
Supplement: S3 Table — (PDF) [file pone.0230550.s003.pdf]

S4 Table. Quantities of PCR components used in this Study.

**Material**	**Volume (ul)**
Sterile DW	7.6
10X PCR buffer (Mg^{2+} 20mM contained)	2.0
dNTP (2.5 mM each)	1.6
$MgCl_2$ (25 mM)	1.0
i-taq polymerase (5 units/ul)	0.3
Template DNA (5 ng/ul)	1.0
L. gasseri forward primer (10 pmole/ul)	0.25
L. gasseri reverse primer (10 pmole/ul)	0.25
L. acidophilus forward primer (10 pmole/ul)	1.0
L. acidophilus reverse primer (10 pmole/ul)	1.0
L. helveticus forward primer (10 pmole/ul)	0.25
L. helveticus reverse primer (10 pmole/ul)	0.25
L. jensenii forward primer (10 pmole/ul)	0.25
L. jensenii reverse primer (10 pmole/ul)	0.25
L. crispatus forward primer (10 pmole/ul)	0.5
L. crispatus reverse primer (10 pmole/ul)	0.5
L. gallinarum forward primer (10 pmole/ul)	1.0
L. gallinarum reverse primer (10 pmole/ul)	1.0
**Total**	**20.0**
