# Supplementary material for: Genome-based species-specific primers for rapid identification of six species of Lactobacillus acidophilus group using multiplex PCR
Source: PLoS One. 2020 Mar 20;15(3):e0230550. doi: 10.1371/journal.pone.0230550 (PMC7083307; doi:10.1371/journal.pone.0230550)

S2 Figure. Phylogenetic tree of the *Lactobacillus* 16S rRNA gene sequences.

Number of bootstrap replications are 1000 and the values are shown on the left branches. Two *Enterococcus* strains, *Escherichia coli* ATCC 11775 and *Salmonella enterica* ATCC 13314 were included as outline. T means type strain.

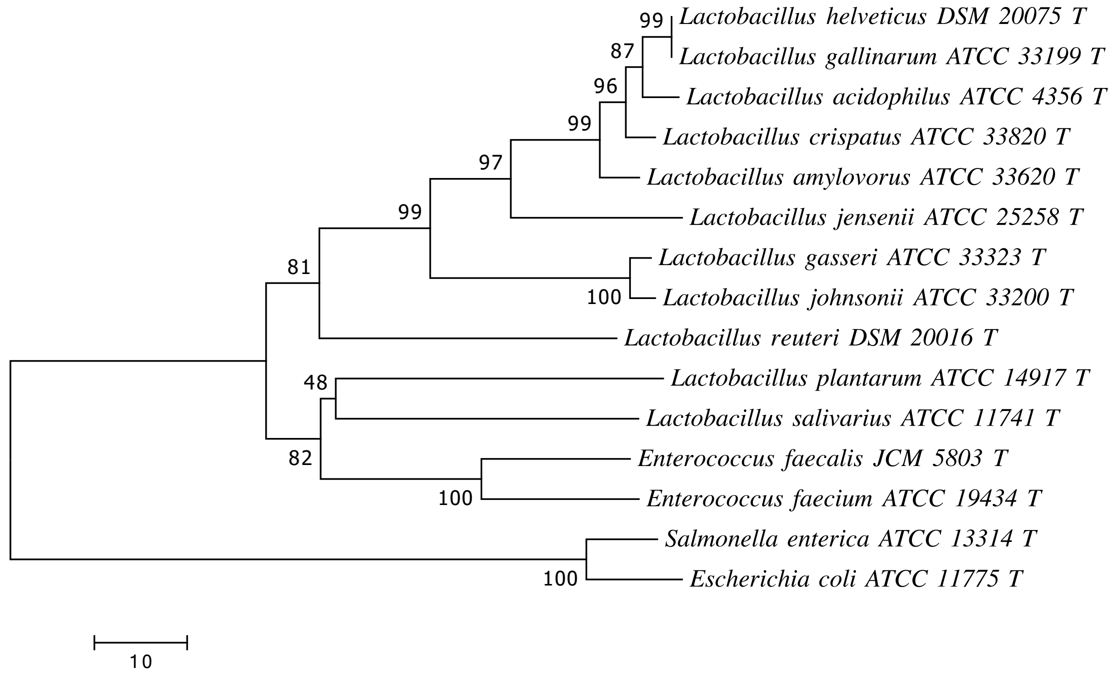

Supplement: S1 Fig — (PDF) [file pone.0230550.s004.pdf]
