# Supplementary material for: Genome-based species-specific primers for rapid identification of six species of Lactobacillus acidophilus group using multiplex PCR
Source: PLoS One. 2020 Mar 20;15(3):e0230550. doi: 10.1371/journal.pone.0230550 (PMC7083307; doi:10.1371/journal.pone.0230550)

S7 Figure. Gel Electrophoresis Results of mixed genomic DNA containing target and untargeted species.

DNA mix was composed of total eleven genomic DNA containing six target species (*Lactobacillus gasseri*, *Lactobacillus acidophilus*, *Lactobacillus helveticus*, *Lactobacillus jensenii*, *Lactobacillus crispatus* and *Lactobacillus gallinarum*) and five untargeted species (*Lactobacillus plantarum*, *Lactobacillus reuteri, Lactobacillus salivarius*, *Enterococcus faecalis* and *Enterococcus faecium*). M is for size marker(bp); N is for negative control.

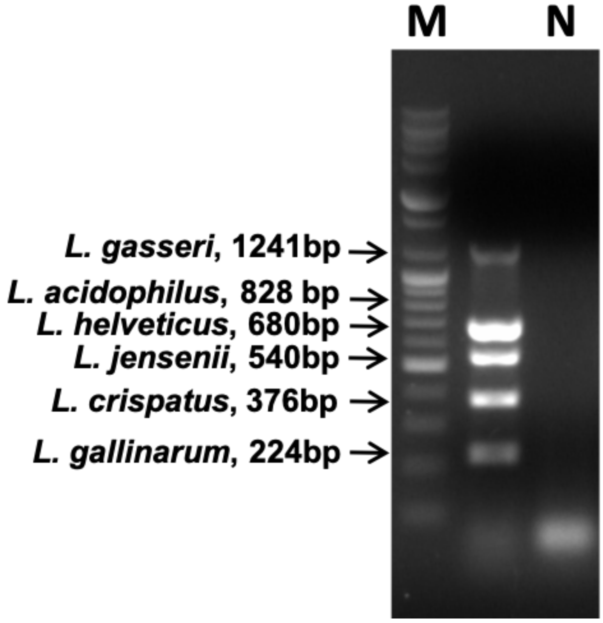

Supplement: S2 Fig — (PDF) [file pone.0230550.s005.pdf]
