# Supplementary material for: Genome-based species-specific primers for rapid identification of six species of Lactobacillus acidophilus group using multiplex PCR
Source: PLoS One. 2020 Mar 20;15(3):e0230550. doi: 10.1371/journal.pone.0230550 (PMC7083307; doi:10.1371/journal.pone.0230550)
Supplement: S1 Data — (PDF) [file pone.0230550.s006.pdf]

S5 Data. Species-specific gene sequences for each species.

>*Lactobacillus gasseri*, Hypothetical protein, 2367 bp

GTGCGGAGGCTTGGTCGTTTGGCAGCCCAACTCACGATACACGCTACTTCAT

CCGCCTTGCCAAACGGAAGAATAAAGAAGTTCGATACTTTAATACAAGAAT

GGAGGATTGCTAAGATGCACTTTACTTTATCAACAGCAGCCAATTCCGGTCA

GGCCAGTAACACTATTTACCCTAACCAGCTAATTATCACTAACTCCCAAGAA

TTACAGCAGGCCGTACAGTATGACCATGTTTGTGGGCGGTTCAAGAATAAC

CAACGTAACATTGCAAACTTCATCAAAGCTGACTGCTTAGTCATGGATTGTG

ACAATGACCACTCTGATGATTCTGCCGCTTGGATTAATCCTACCAGCATTGC

GAATTACTTTGATGGCGTTTCTTATGCCATTACATTATCGCGCAACAACATG

AAAGCTAAGAACAATAAAGCCCCGCGCCCAAAGTTCCACATCTACTTTCCA

ATTAGCGAGATCAATAATGCTAAAACCTACGCTGAATTAAAACATGAAATT

CAAGAATATTTCCCCTATTTTGATAACAATGCTCTCGATGCTGCCCGCTTCG

TATTTGGTGTTCCCAGTACACAAGTTAGTTGGCACGAAGGATCCCAAACCAT

CGACCAATTTATGATGGCGCAACGTTACTTTGCCAAGCAAAAGGTCGGATC

GATTCGCCAAGGCAAACGCAATGCAACTCTCTCCCACTTTGCTGGTCGAATC

ATTATGCGATTGGGCAATACTCCCGAAGCACGTCAGGCCTTTCAAGACGAG

GCAGCGAAGTGTGAACCGCCACTGGGAGAACAAGAACTAAGAACCATCTG

GCATAGTGCTATCAAATTCGGCCACCGAATGGCCAGTAAAAAAGGTTATAT

TCCACCTGAAGAATATAATCAACCCAATGATGATCTGCATCCCTACGATTAT

TCGGATACTGGCGAGTCCTATGTTTTTGTTAACAACTGTAAGGACCGCGTCT

GCTACACCAACCAGTCAGGTTTTATGTGGTTTGACGGTAAAATTTGGCAAGA

ATCGGAGCCCCTCGCTCTCGGCGAGGTTCAACGCTTTACTGACAAACAACTT

GCGGATGCTCAACTACGAGTCACTAAGGCTTACCAAGTGATCCAACAAAAT

GGCGTAACTAGCGCGCTTCAAACGATGGGCAAAACGAAGGCTAGTCGCACT

TTTAATGATGATCAGCAAGCTACGTTCAAAGAATATCAAAACGCCAAGGCT

TATGAAGCTTTTATTCTCAAGGAACGGAGCACCCGTGGCATCAATGGAATCT

TGACTAACGCCCGGCCAAAGTTAGTAAAAGAAATCAATGAATTCGATGCTA

ATCCCTTTTTATTAAACACTCCTGATGGCCCTTACAACCTTAAACAGGGCAT

TCATGGGCAACAAGAAATTCAAGCCAGCGATTTGATTACTAAGTCCACGTC

TTGTGTGCCTGGCAGTCAAGGAAATTCAATCTGGCAAGAAGCCCTAAATAC

ATTCTTTTGTAACGACCTAGCACTAATAAATTACGTTCAAGAAATTGTGGGA

CTCGTTGCCATTGGTCAGGTTTACTTGGAAGCGTTGATTATTGCATATGGCA

GTGGACGAAATGGTAAATCCACTTTCTGGAACACAATTGCCAATGTACTCG

GTTCTTATACTGGTCACCTCTCAGCTGATGCCTTAACAACAGGTGTTCGACG

GAATGTCAAACCAGAAATGGCTGAAGTCAAAGGTAAACGCTTAATCATCTC

TGCTGAGCTGGAAGAAGGCAAACGACTAAACACTTCGATTGTCAAACAACT

CTGTTCAACTGATGAAATCTACGCTGAGAAAAAATACATGAAGCCCTTCTCT

TTTACACCTAGTCATACCATCGTTCTCTATACCAACTACCTGCCTCACGTGG

GCGGTAACGATGAAGGAATCTGGCGGCGATTGATCGTAATACCGTTTAAAG

CCACGATTGCCAAACACAATGATATTAAAAATTATGCCCAGTACCTAACCG

AACAAGCCGGTCCGGCAGTGTTGCAATGGATCATTGAGGGCGCGCAACGAA

TCATTCAACAAAATTACCAGCTAACTACTCCGGCAGCAGTTACTAAAGCAG

TCAAGGACTACCACGCCGATAATGACTGGTTAGGTCATTTTCTCAATGAAAA

TTGCGAACTTGATTCTAGTTATCAGCAAAAATCGGGTGACCTCTATCAAAAG

TACCGAGAATACTGCCAAGGTATCGGCGAATACACCCGAAGTACCACTGAC

TTTTACACGGCCCTTAAGAATGCGGGCTTTCAACGTCAACGTAAGAATACTG

GATCCTATGTTCGCGGACTGCGCTTAAAGGCATCAGAATTTCTCGACTAG

>*Lactobacillus acidophilus*, MRS- transporter, 959 bp

TTGCATATTTTGACGGCGATGTTCGTGCTGATTGCGTTCATGTTGGGATGCA

ATGAGTTTATGGTAGTCGGAAATTTGTCACTGATTGCGCAAACTTATCATGA

ATCGCTGAGTCAGATTTCTTGGTTGGTATCGGCGTTTGCATGGACTTATGCG

ATCGTGACACCGCTCCTGGCTTTATTTACCAATAAGATTCACAAATACTATT

TGTTGATTTTTTTGACGGGAACAATTTTGAGCTCGTGTGCACCAAGTATTGG

TTGGCTGCTTTTTTCAAGAATTATTACAGCATCAGTGGCAGGAATGATTGAA

TCACTGCTATCGGTAATCGTTTACCAGATATTGCATAATCAAAAATAGCGCT

CGGTGACGATTGTCTGGATTTATACAGGCTTTAGCATCGGTTGTGGGTGTGC

CGCTGGGAACGGTCATTGCTGATCACTGGCGATGGCAGGATGCTTTCACCAT

GTGTGTGGTAATTACAGCGGTGGCTACAATTATTGCGTTGCTAGTTTTGCCT

AAAAACTTGAATGCAGGTGAGGGTAATTATAGCGATCGGATTCAGATTTTC

AAGGATAAAACAATCTGGTATGGGATCGGCTTCGTTATCTGTGCGGCAGCT

ACCTTGTATGGCTATTACACGTATATTAGACCGCTGGTTCATGTGCAACTTA

AGTTCGATTTGAATGCATTGAGTTTGATTTGGCTACTCCTTGGTGTGGTAGC

TATTTTTGGCTCGACAACACAAGTATTATTTTTGAATGAAGCATCGAAAAAA

TATCCGGCAGCGATTAGTTTGGCATCAACGTTAAGTGCAATTTTTTATAATG

TCGGTATCTTCAATGACAGCAGGACAAGTTTTGAAATACGGTGGTTTAACTA

GTTTGGGCTGGAATTCATTTGTTTATTGCTTTGTTTTGGCTAGAAAACTAGAT

AATAGAAATTTGGGGGATAAGTAG

>*Lactobacillus helveticus*, ACPS-malonyltransferase, 918 bp

TTATTTCACCAACTTTGCTTTTGATCGAAAATCGTTTAACGTCTTAACACTAT
CAATATGGAATGTATGATCGTTCGCCACCACATTCTTGGCAAACTTCATCAA
CGTATCGCCAGGGCCCAGTTCAACAACTGTATCAACACCCAATTGAGTAAG
CTGTTGAATGCAGTTATAAAAATGGGTTGGATTAATGAGCTGATCGATTAAC
GTTTGCTTAATCGTGTTAACTTCGAAAGGTTGAGACGTTGTGTTACTGATCA
CTGGGAAAGCCAACTGGTTAAACGAGACATCTTGAATTCGTTTTGCCAACA
AATCGGAGGCCTCCTGCATAAATGGGGTGTGAGACGCAACCGTCATTTTCA
ACGGGACAACCCGTTTAACACCGTGCTCATGCAGATAATCGGTAGCTGCTT
GAAGCCCCTCAATGGAACCACCAATCACAATTTGAGAATCTGTGTTGTAATT
GGCTACATAGATCTCACCCTTCTTCGAACCGACTTTTACCGCTTGGTCGACC
ATGTCAGCAGTCGTTTTTAAGACAGCCGCCATTTTGCCAGGATGATCCTGAC
CGGCTTTATCCATGTAGTGACTGCGATCACGAACCAATTGAAGGGCATCACT
GAAGTCCAATCCCTTGGCAGCGACAATCGCGCTATACTCACCAAGACTCAA
ACCAGTGGCACCAACTGGATCACCAAAATCTTGATTGATAATCCGTTCAATC
CCTGTACTCATGGCGACAATCGCCACTTGGGTATTGACCGGATTATCAAAAA
CGGTGGCATCGCTCATATCCATATTCAAAGCTTCGGAAGCTTGATCAATTGT
TTGCCGATAAACAGGTTCTTGTTGATACAAGTCCTGTCCCATTTTGCCAAAT
TGCTTACCTTGGCCGCTAAATAAATAACCTAGTTTCAA

>*Lactobacillus jensenii*, Acetoacetate decarboxylase, 828 bp

ATGACTAGTTATCTTGTTAACAAAGAAGATGTTGTCAACTTTCTTGATAATC
CTAACATGAAAAATCAAGATGGTTATATGTTTGCCTACGTCACTTCACCAGA
AGTTTTAGCTAGATTAGTGCCTAAGCCATTAACGCCTATCGCACCAGTCATC
GCTGGTTATATTACCCATATGGGCAATCCAACTTTTGCTAAGCCTTATGATG

AAGCTGTCTTATATAGTTTAGTTTCATACGGTGGCCGCATGCTTGGCGCTTA

TCCTTTCACCCTATTTTTAAGTGGTGAAGGAGCCGAAGAAGCCATGCTTGCT

GGTCGTGAAGGAGCTGCAATTCCAAAGAAGTTAGCTGATAAAATTAGCTAC

ACTGAAAAAAATGGCAGCACCCATGTTGTTGTTACTAGACATGGTGTAGAT

TTAATTAATCTTACCTTTACTCCAGGTATTCCCAACGATCCTGACATCGCTA

AACAGTTAATGGGTGGTCAATCTAAGCTTGATACGCCAACCGATACTTATA

GTTTTTTCTTCGACTATAAGATTGATCAAGACCATGACGGTCACAACCACTT

CATTAACACTCGTTTAATTGCTACTAAGACAACAGGTGTCACCCACGCATTC

ACTCCAGGCAACATTACAATGGAACTTGGGACAAGTAGCGATGATCCAGTC

AGCGAATTAACCGTATTAAAGCCAGTTGGTGGCGCTCACTACCAAATGACT

AGTGGCAAAATGCACGAAACAATTCAACTTGCCCAAGTTGAACCAGATGAA

ATCGCACCATATTTAATTACTGGCCGCTATGACCAAGCTATTTTAGGAAAGT

AA

>*Lactobacillus crispatus*, Hypothetical protein, 420 bp

ATGGCGAAGAGACACCAATATCTTTGGTGTTTGGTTGAATTGCCTAACGGCA

AACGCGAATGGTACTGCATTTCTAAGGTATTGCGCAAGGCCTTACTTTGGGA

GAAAAATTATCTGCATAATCGATACTGGCGCAATACTTTAATTGGTAGCTAC

CTCAATGTTGCTCGCACGCGTTATCATCATGATCGGGCAATTATTACAGTAG

GAAGGGTAATCCGAGTGAAAATTTTGTATTATCCTACTCAGGATTGGCATTG

GACACGCAATCAATTTATCGCGGCGGGACAATTGGATAACTTTGCCACCGC

CTATAATTATATGAAGCACAATTATGCTTGGTACAACAAGCTATTGATTCAT

CATGCGTTACGTCATTGGCGTAGAATAAGCACTTCTAAACATTGCAATAAAT

TCTAA

> *Lactobacillus gallinarum*, Hypothetical protein, 552 bp

ATGATTCTAGAGCTTTTTATTTTGTTAAATTGTTTAACTCTAAAGGTATCATA

TTATAATCTTAAAGAAAGAGACCACATCATTATAAGTAATTCTCTTAGCATA

ATTTCTAAACATCACCAAGGAGTACCATATCTGAAGATGAAAACTAAAATA

ATATCAATAATACTTTTAATACTCATCGTCCCACCTATTCTTTTCTGTGTACT

TAATTCTAGTTACGAGTTATACGTTAATCATCAAGAAAATATTGCATACAGT

AAAATGCAACAAGCTGTAGACAAACATGGCTATAATGCAATTAGTCTTGAG

CCCGTAAAAGCTCGAATAAAATTCTTTCACATTTATGAATATAAAAGTTCAT

TTTCTAATCAAAAAACATTGAATAAGAATAGACAACTCTTCAACAAAGTTG

GATATAAGATAGGCAAACATGATGATCTAGAAACGCCTTATAAATATTCAA

TCAGTGCCCTAAAATCAAACGAAAAATGGACAGTTTATATTAAAGAAGAAC

CGTTTGGCAAAGAAAAAAGTTATCGCGAAGATTGA
