# Supplementary material for: Genome-based species-specific primers for rapid identification of six species of Lactobacillus acidophilus group using multiplex PCR
Source: PLoS One. 2020 Mar 20;15(3):e0230550. doi: 10.1371/journal.pone.0230550 (PMC7083307; doi:10.1371/journal.pone.0230550)
Supplement: S2 Data — (PDF) [file pone.0230550.s007.pdf]

S6 Data. Sequences of PCR products of each species.

> *Lactobacillus gasseri* 1107

AGAACCATCTGGCATAGTGCTATCAAATTCGGCCACCGAATGGCCAGTAAAA
AAGGTTATATTCCACCTGAAGAATATAATCAACCCAATGATGATCTGCATCCCT
ACGATTATTCGGATACTGGCGAGTCCTATGTTTTTGTTAACAACTGTAAGGAC
CGCGTCTGCTACACCAACCAGTCAGGTTTTATGTGGTTTGACGGTAAAATTTG
GCAAGAATCGGAGCCCCTCGCTCTCGGCGAGGTTCAACGCTTTACTGACAAA
CAACTTGCGGATGCTCAACTACGAGTCACTAAGGCTTACCAAGTGATCCAAC
AAAATGGCGTAACTAGCGCGCTTCAAACGATGGGCAAAACGAAGGCTAGTC
GCACTTTTAATGATGATCAGCAAGCTACGTTCAAAGAATATCAAAACGCCAA
GGCTTATGAAGCTTTTATTCTCAAGGAACGGAGCACCCGTGGCATCAATGGA
ATCTTGACTAACGCCCGGCCAAAGTTAGTAAAAGAAATCAATGAATTCGATG
CTAATCCCTTTTTATTAAACACTCCTGATGGCCCTTACAACCTTAAACAGGGC
ATTCATGGGCAACAAGAAATTCAAGCCAGCGATTTGATTACTAAGTCCACGT
CTTGTGTGCCTGGCAGTCAAGGAAATTCAATCTGGCAAGAAGCCCTAAATAC
ATTCTTTTGTAACGACCTAGCACTAATAAATTACGTTCAAGAAATTGTGGGAC
TCGTTGCCATTGGTCAGGTTTACTTGGAAGCGTTGATTATTGCATATGGCAGT
GGACGAAATGGTAAATCCACTTTCTGGAACACAATTGCCAATGTACTCGGTT
CTTATACTGGTCACCTCTCAGCTGATGCCTTAACAACAGGTGTTCGACGGAAT
GTCAAACCAGAAATGGCTGAAGTCAAAGGTAAACGCTTAATCATCTCTGCTG
AGCTGGAAGAAGGCAAACGACTAAACACTTCGATTGTCAAACAACTCTGTT
CAACTGATGAAATCTACGCTGAGAAAAAATACATGAAGCCCTTCTCTTTTACA
CCTAGTCATACCATCGTTCTCTATACCAACTACCTGCCTCACGTGGGCGGTAA
CGATGAA

> *Lactobacillus acidophilus* 756

TGAGTTTATGGTAGTCGGAAATTTGTCACTGATTGCGCAAACTTATCATGAAT

CGCTGAGTCAGATTTCTTGGTTGGTATCGGCGTTTGCATGGACTTATGCGATC

GTGACACCGCTCCTGGCTTTATTTACCAATAAGATTCACAAATACTATTTGTTG

ATTTTTTTGACGGGAACAATTTTGAGCTCGTGTGCACCAAGTATTGGTTGGCT

GCTTTTTTCAAGAATTATTACAGCATCAGTGGCAGGAATGATTGAATCACTGC

TATCGGTAATCGTTTACCAGATATTGCATAATCAAAAATAGCGCTCGGTGACG

ATTGTCTGGATTTATACAGGCTTTAGCATCGGTTGTGGGTGTGCCGCTGGGAA

CGGTCATTGCTGATCACTGGCGATGGCAGGATGCTTTCACCATGTGTGTGGTA

ATTACAGCGGTGGCTACAATTATTGCGTTGCTAGTTTTGCCTAAAAACTTGAA

TGCAGGTGAGGGTAATTATAGCGATCGGATTCAGATTTTCAAGGATAAAACAA

TCTGGTATGGGATCGGCTTCGTTATCTGTGCGGCAGCTACCTTGTATGGCTATT

ACACGTATATTAGACCGCTGGTTCATGTGCAACTTAAGTTCGATTTGAATGCA

TTGAGTTTGATTTGGCTACTCCTTGGTGTGGTAGCTATTTTTGGCTCGACAAC

ACAAGTATTATTTTTGAATGAAGCATCGAAAAAATATCCGGCAGCGATTAGTT

TGGCATCAACGT

> *Lactobacillus helveticus* 643

TCTTGGCAAACTTCATCAACGTATCGCCAGGGCCCAGTTCAACAACTGTATC

AACACCCAATTGAGTAAGCTGTTGAATGCAGTTATAAAAATGGGTTGGATTAA

TGAGCTGATCGATTAACGTTTGCTTAATCGTGTTAACTTCGAAAGGTTGAGAC

GTTGTGTTACTGATCACTGGGAAAGCCAACTGGTTAAACGAGACATCTTGAA

TTCGTTTTGCCAACAAATCGGAGGCCTCCTGCATAAATGGGGTGTGAGACGC

AACCGTCATTTTCAACGGGACAACCCGTTTAACACCGTGCTCATGCAGATAAT

CGGTAGCTGCTTGAAGCCCCTCAATGGAACCACCAATCACAATTTGAGAATC

TGTGTTGTAATTGGCTACATAGATCTCACCCTTCTTCGAACCGACTTTTACCGC

TTGGTCGACCATGTCAGCAGTCGTTTTTAAGACAGCCGCCATTTTGCCAGGAT

GATCCTGACCGGCTTTATCCATGTAGTGACTGCGATCACGAACCAATTGAAGG

GCATCACTGAAGTCCAATCCCTTGGCAGCGACAATCGCGCTATACTCACCAA

GACTCAAACCAGTGGCACCAACTGGATCACCAAAATCTTGATTGATAATCCG

TTCAATCCTGAC

> *Lactobacillus jensenii* 510

CCTTTCACCCTATTTTTAAGTGGTGAAGGAGCCGAAGAAGCCATGCTTGCTG

GTCGTGAAGGACTGCAATTCCAAAGAAGTTAGCTGATAAAATTAGCTACACT

GAAAAAAATGGCAGCACCCATGTTGTTGTTACTAGACATGGTGTAGATTTAAT

TAATCTTACCTTTACTCCAGGTATTCCCAACGATCCTGACATCGCTAAACAGTT

AATGGGTGGTCAATCTAAGCTTGATACGCCAACCGATACTTATAGTTTTTTCTT

CGACTATAAGATTGATCAAGACCATGACGGTCACAACCACTTCATTAACACTC

GTTTAATTGCTACTAAGACAACAGGTGTCACCCACGCATTCACTCCAGGCAA

CATTACAATGGAACTTGGGACAAGTAGCGATGATCCAGTCAGCGAATTAACC

GTATTAAAGCCAGTTGGTGGCGCTCACTACCAAATGACTAGTGGCAAAATGC

ACGAAACAATTCAACTTGCCCAAGTTGAACCAGAT

> *Lactobacillus crispatus* 348

ATATCTTTGGTGTTTGGTTGAATTGCCTAACGGCAAACGCGAATGGTACTGCA

TTTCTAAGGTATTGCGCAAGGCCTTGCTTTGGGAGAAAAATTATCTGCATAAT

CGATACTGGCGCAATACTTTAATTGGTAGCTACCTCAATGTTGCTCGCACGCG

TTATCATCATGATCAGGCAATTATTACAGTAGGAAGGGTAATCCGAGTGAAAA

TTTTATATTATCCTACTCAGGATTGGCATTGGACACGCAATCAATTCATTGCGG

CTAGCCAACTGGAAAATTTTACAACCGCCTATAATTATATGAAGCACAATTATG

CTTGGTATAACAAGCTATTGATTCATCA

> *Lactobacillus gallinarum* 578

GTTATTGGTTTTTTTACCCCTGGGAGGATTAGGTTTTTTTTGGTGTTTATTCTTT

CCGTTCGACCCTGTGCCCGGGTTGAAGGCGAGAGCCCCCCCCAGCCCCCCC

ATCGTGATACCAGTGGGAGGCCCCCAAACAAACAGAAGGAAGGGTGATTTC

ATTCCCCCCGGTTTTCGGAAAAACGGTCCAGGTTACCGCCAAAAGTTTTGAC

GGAAAAGGGGGAGGATCCAATAGCTTGTTATACCGAGCGAAACTAAACCTCC

ATATATTATAGGCGGTTGTAAAATTTTCCTGTCGGCTAGCCGCACTGAATTGAT

TGCGTGTCCAATGCCCTTCCATGAGTAGGATAATTTTTTTTTTTCGGTGTCTTG

AGCCCCGTAAAAGCTCGAATAAAATTCTTTCACATTTATGAATATAAAAGTTC

ATTTTCTAATCAAAAAACCTTGAATAAGAATAGACAACTCTTCAACAAAGTT

GGATATAAGATAGGCAAACATGATGATCTAGAAACGCCTTATAAATATTCAATC

AGTGCCCTAAAATCAAACGAAAAATGGACAGTTTATATTAAAGAAGACCC
